# Relevance of Baseline Viral Genetic Heterogeneity and Host Factors for Treatment Outcome Prediction in Hepatitis C Virus 1b-Infected Patients

**DOI:** 10.1371/journal.pone.0072600

**Published:** 2013-08-28

**Authors:** Verónica Saludes, Elisabet Bascuñana, Elena Jordana-Lluch, Sònia Casanovas, Mercè Ardèvol, Esther Soler, Ramón Planas, Vicente Ausina, Elisa Martró

**Affiliations:** 1 Microbiology Service, Fundació Institut d’Investigació en Ciències de la Salut Germans Trias i Pujol, Hospital Universitari Germans Trias i Pujol, Universitat Autònoma de Barcelona, Badalona, Spain; 2 CIBER Epidemiología y Salud Pública (CIBERESP), Barcelona, Spain; 3 Hospital Pharmacy, Hospital Universitari Germans Trias i Pujol, Badalona, Spain; 4 Liver Unit, Hospital Universitari Germans Trias i Pujol, Badalona, Spain; 5 CIBER Enfermedades Hepáticas y Digestivas (CIBEREHD), Barcelona, Spain; 6 CIBER Enfermedades Respiratorias (CIBERES), Bunyola, Spain; Temple University School of Medicine, United States of America

## Abstract

**Background:**

Only about 50% of patients chronically infected with HCV genotype 1 (HCV-1) respond to treatment with pegylated interferon-alfa and ribavirin (dual therapy), and protease inhibitors have to be administered together with these drugs increasing costs and side-effects. We aimed to develop a predictive model of treatment response based on a combination of baseline clinical and viral parameters.

**Methodology:**

Seventy-four patients chronically infected with HCV-1b and treated with dual therapy were studied (53 retrospectively −training group−, and 21 prospectively −validation group−). Host and viral-related factors (viral load, and genetic variability in the E1–E2, core and Interferon Sensitivity Determining Region) were assessed. Multivariate discriminant analysis and decision tree analysis were used to develop predictive models on the training group, which were then validated in the validation group.

**Principal Findings:**

A multivariate discriminant predictive model was generated including the following variables in decreasing order of significance: the number of viral variants in the E1–E2 region, an amino acid substitution pattern in the viral core region, the *IL28B* polymorphism, serum GGT and ALT levels, and viral load. Using this model treatment outcome was accurately predicted in the training group (AUROC = 0.9444; 96.3% specificity, 94.7% PPV, 75% sensitivity, 81% NPV), and the accuracy remained high in the validation group (AUROC = 0.8148, 88.9% specificity, 90.0% PPV, 75.0% sensitivity, 72.7% NPV). A second model was obtained by a decision tree analysis and showed a similarly high accuracy in the training group but a worse reproducibility in the validation group (AUROC = 0.9072 *vs*. 0.7361, respectively).

**Conclusions and Significance:**

The baseline predictive models obtained including both host and viral variables had a high positive predictive value in our population of Spanish HCV-1b treatment naïve patients. Accurately identifying those patients that would respond to the dual therapy could help reducing implementation costs and additional side effects of new treatment regimens.

## Introduction

Hepatitis C virus (HCV), with an estimated 150 million people chronically infected worldwide, is the major causative agent of chronic liver disease, cirrhosis and hepatocellular carcinoma [1]. HCV has a positive single-stranded RNA genome that exhibits significant genetic variability, leading to the circulation within an infected individual of a dynamic mosaic of closely related viral variants usually referred to as quasispecies. This phenomenon has been associated with chronic infection establishment, pathogenicity and resistance to antiviral drugs [2].

Pegylated-interferon alpha (PegIFN-α) and ribavirin (RBV) combination therapy constitutes the current standard of care for the treatment of chronic hepatitis C by non-1 genotypes [3]. However, triple therapy adding an HCV-specific protease inhibitor (PI) [4], [5] has recently been approved for chronic infection by HCV genotype 1 (HCV-1) in several countries in America, Europe, the Middle East, Asia and Australia. Treatment failure rates for naïve HCV-1-infected patients decrease from 40–50% with PegIFN-α and RBV [6], [7], to about 25–33% with the triple therapy [4], [5], [8]. However, the addition of a PI increases the costs, side effects and drug-drug interactions of the dual therapy, and the efficacy of triple therapy depends largely on susceptibility to PegIFN-α and RBV. Therefore, there remains a need to identify those patients most likely to respond to this dual therapy before starting treatment in order to decrease the implementation costs of novel triple therapies, as well as the additional side effects. This will constitute a step forward towards personalized treatment regimens of chronic hepatitis C.

A number of host-related factors have been associated with IFN-α-based treatment failure in HCV-1-infected patients, such as African-American ancestry, advanced liver fibrosis or cirrhosis, older age, male gender, metabolic disorders, transaminase levels and, more recently, several host genetic polymorphisms and the level of certain chemokines in serum (reviewed in [9] and [10]). Baseline virological factors include high viral loads, high levels of genetic variability within the E1–E2 and NS5A regions, as well as mutations in the so-called Interferon Sensitivity Determining Region (ISDR) and the core region [11], [12]. Nevertheless, such associations have not always been found and remain controversial.

Although most studies focussed on the prediction of treatment outcome have been based on the predictive value of single host or viral factors, more recently predictive models combining several variables have been proposed for chronic infection by HCV-1. However, most of these models have not been validated and/or have a variable accuracy (reviewed in [13]). We previously developed a predictive model based on baseline host and viral variables [14]. In the current study, we considered additional variables including the *IL28B* polymorphism, and increased the sample size used to generate and validate new predictive models of PegIFN-α and RBV therapy response in HCV-1b infected patients.

## Materials and Methods

### Ethics Statement

This study was approved by the Clinical Research Ethics Committee at our institution (“Comité Ético de Investigación Clínica”, CEIC). Written informed consent was obtained for all patients.

### Patients and Specimens

A total of 74 patients with chronic hepatitis C by HCV-1b treated with combination therapy at “Hospital Universitari Germans Trias i Pujol” were included. All of them were Caucasian and of Spanish origin. Exclusion criteria were: previous IFN-α-based treatment, HIV or HBV coinfection, alcohol abuse or having other causes of chronic liver disease. The patients had started antiviral therapy with PegIFN-α2a (180 µg/week) plus weight-based doses of RBV (1000–1200 mg/day) for 48 weeks between 2003 and 2011. The patients were considered either as responders (SVR, defined as undetectable HCV-RNA in serum 24 weeks after treatment cessation) or non-responders (continued presence of HCV-RNA during therapy −null response−, rebound of HCV-RNA while on therapy −breakthrough−, or 24 weeks after the end of treatment −relapse−). Included patients were classified into two groups: the training group consisted of 53 patients (retrospective study) and the validation group included 21 patients (prospective study). All virological analyses were performed using serum specimens obtained before treatment initiation and conserved at −80°C until testing.

### Baseline Clinical and Epidemiological Host Parameters

The following variables were obtained by clinical record review: gender, age, weight, body mass index (BMI), stage of fibrosis according to the Forns index [15], serum levels of cholesterol, platelets, ALT, AST, and GGT. Enzyme levels were transformed into a quotient expressing the factor times upper limit of normal (ULN) according to gender. A good treatment adherence was considered when having received ≥80% of total maximum dose prescribed of both drugs for ≥80% of the expected duration of therapy [16].

Besides, the *rs*12979860 polymorphism near the human *IL28B* gene was determined by real-time PCR using the LightMix® Kit *IL28B* (Roche Diagnostics GmbH, Mannheim, Germany) according to manufacturer’s instructions starting from whole blood specimens collected in tubes containing EDTA.

Finally, serum levels of human Interferon-γ Inducible Protein 10 (IP-10) were quantitatively determined with the Quantikine ELISA Human CXCL10/IP-10 Immunoassay (R&D Systems, Abingdon, UK), following manufacturer’s instructions. Patients were classified as having low or high IP-10 values using a 600 pg/mL cut-off [17].

### Baseline viral Parameters

#### Serum viral load

HCV-RNA had been quantified by RT-PCR (Cobas® Amplicor HCV Monitor test, Roche Molecular Systems, Pleasanton, CA, USA) or by real-time RT-PCR (Abbott RealTi*m*e HCV assay, Abbott Molecular Inc., Des Plaines, IL, USA), according to manufacturer’s instructions and was recorded as Log_10_IU/mL.

#### RNA extraction and reverse transcription

Total RNA was extracted from 220 µl of serum, using the QIAamp® viral RNA kit (QIAGEN® GmbH, Hilden, Germany) according to the manufacturer’s protocol. Reverse transcription was performed using random hexamers in order to prevent any bias during the reaction, as previously described [18].

#### PCR-cloning and sequencing of the E1–E2 region

A 532-bp sequence encompassing the E1 C-terminal and the E2 N-terminal regions (including the HVR-1, HVR-2 and HVR-3) was amplified, cloned and sequenced as previously described [14] and referred to as E1–E2 region (nucleotides 1322–1853 in the H77 reference sequence, GenBank accession number AF009606). For the prospective patients, E1–E2 PCR products were cloned using the Zero Blunt TOPO PCR cloning kit for sequencing (Invitrogen). Between 24 and 35 colonies were selected and subjected to PCR followed by purification and sequencing of both DNA strands. Sequence readings were assembled and edited with the STADEN package v1.6. [19].

#### PCR and direct sequencing of the core region

The whole core region (573 bp, H77 positions 342–914) was amplified and sequenced as previously described [14]. Sequences were assessed for the presence of amino acid polymorphisms associated with treatment outcome.

#### PCR and direct sequencing of the NS5A region

A fragment of the NS5A region containing the ISDR was amplified and directly sequenced as described by Torres-Puente *et al*. [20]. The number of amino acid substitutions with respect to the HCV-J strain was determined.

#### Phylogenetic analysis of the core and E1–E2 regions

Phylogenetic analysis of the HCV core region derived from the patients included in the study together with reference sequences was used to confirm that the HCV genotype and subtype was 1b. E1–E2 sequences were also subjected to phylogenetic analysis in order to rule out potential contamination between specimens. Sequences were aligned by ClustalW implemented in MEGA 4 [21]. Maximum-likelihood phylogenetic trees were obtained with PHYML [22].

#### Genetic variability analysis of the E1–E2 region

Multiple alignments were generated with all clones generated from each patient for the complete E1–E2 region, and the HVR-1, HVR-2 and HVR-3 subregions (H77 nucleotide positions 1491–1571, 1761–1787, and 1632–1739, respectively). The following genetic variability estimates were obtained for each multiple alignment with DnaSP v4.50 [23]: total number of polymorphic sites (S), total number of mutations (η), nucleotide diversity corrected by Jukes-Cantor method (π), and number of viral variants (number of haplotypes, nHap). The number of substitutions per synonymous site (Ks) and number of nonsynonymous substitutions per nonsynonymous site (Ka) were obtained using the Nei-Gojobori method.

#### Statistical analysis

Clinical and virological values were compared between responders and non-responders in bivariate analysis. Student’s *t* test (Normal distribution) or Mann-Whitney *U* test (non-Normal distribution) were used for quantitative variables, and Chi-square, Fisher’s exact test or Likelihood ratio test were used for categorical variables. Data was expressed as mean ± standard deviation, median and range, or relative frequency.

Statistical models were developed to predict treatment outcome. Firstly, a multivariate discriminant analysis [24] was carried out to develop a predictive model on the training group, which was then validated in the validation group. Covariates initially included in the discriminant model were explanatory variables that achieved a *p*-value ≤0.15 in the bivariate analyses (training group). Some variables were transformed (square root) in order to achieve normality. The discriminant function was obtained using a backward stepwise variable selection procedure. Secondly, a regression tree analysis [25] was performed using the same initial variables as in the discriminant analysis. The JMP10 software (SAS Institute Inc., Cary, NC, USA) was used to choose the variable and its optimal cut-off that was able to generate the most significant division of the training group of patients into two prognostic subgroups that were as homogeneous as possible for the probability of SVR. Then, this process was repeated on each subgroup of patients in a step-wise manner until no additional significant variable was identified. A ROC curve was obtained for each model and the effectiveness of prediction was measured by: area under the ROC curve (AUROC), sensitivity (proportion of responders which are correctly identified), specificity (proportion of non-responders which are correctly identified), negative predictive value (NPV) and positive predictive value (PPV). Cut-off values that yielded highest PPV and specificity were selected from the ROC curve. The reproducibility of the models was tested with the data from the validation group of patients. Statistical analyses were performed using SPSS v15.0 and JMP10. *P*-values<0.05 were considered significant.

#### Accession numbers

All sequences obtained in this study were deposited in the EMBL Nucleotide Sequence Database (http://www.ebi.ac.uk/embl/) under the following accession numbers: FN675941–FN675983 for core, FN675984–FN676976 and HF572064–HF572784 for E1–E2 and NS5A regions.

## Results

### Patient Groups and Treatment Adherence

The training group consisted of 53 patients with a 47% SVR rate, and the validation group included 21 patients with a 57% SVR rate, adding up to a total of 74 patients. Both patient groups were comparable in terms of descriptive clinical-epidemiological characteristics (**Table 1**). All patients were on treatment for the complete expected time and adherence to both drugs was overall >80%.

**Table 1:** Descriptive baseline clinical features of study patients.

Variable	Training group(N = 53)	Validation group (N = 21)
Age (years)	48.53±11.25	48.52±9.98
Weight (Kg)	75.05±13.73	77.24±11.94
BMI (Kg/m^2^)	26.26 (18.7–41.0)	27.40 (20.8–37.0)
Gender (% male)	29±54.7	11±52.4
ALT quotient (×ULN)	1.68±4.76	1.24±6.34
AST quotient (×ULN)	1.55±3.14	1.30±4.86
GGT quotient (×ULN)	0.81±0.59	0.60±0.63
Patelet count (×10^4^/µl)	164.80±29.36	156.86±27.94
Forns index	5.89±1.69	4.58±2.91
SVR (%)	47.2	57.1
Viral load (Log_10_IU/mL)	5.96±0.70	6.22±0.65

BMI, body mass index; ALT, alanine transaminase; AST, aspartate transaminase; GGT, gamma-glutamyl transferase; ×ULN, factor times upper limit of normal used in our center for males and females: 41 and 31 U/L for ALT, 37 and 31 for AST, and 85 and 50 for GGT, respectively. Data is presented as mean ± SD for variables following a Normal distribution and as median (range) for the rest.

In order to develop predictive multivariate models, firstly, the association between each baseline variable and treatment outcome was studied in the training group of patients.

### Baseline Clinical variables Associated with Treatment Outcome

Baseline clinical characteristics of patients associated with treatment outcome in the training group are shown in **Table 2**. The *IL28B* polymorphism was the variable most strongly associated with treatment outcome (*p* = 1.53×10^−4^) with only 1/13 patients with the favourable C/C genotype not responding to therapy. The AST/ALT ratio (*p* = 0.022) and the GGT quotient (*p* = 0.055) were higher in non-responders, while the ALT quotient was higher in responders (*p* = 0.028). Non-responder patients tended to have a higher Forns fibrosis index score. Both groups of patients were comparable for the rest of variables. Regarding the IP-10 levels, although two patients had insufficient serum volume left to perform the assay, non-responders tended to have high levels of this chemokine more frequently than responders (7/20, 38.1% *vs*. 8/21, 35.0%), but this difference did not reach statistical significance (**Figure S1**).

**Table 2:** Baseline variables used for model development in the training group (*p*-value <0.15).

		Responders (N = 25)	Non-responders (N = 28)	p-value
**Host factors**				
*IL28B* rs12979860 genotype C/C, N (%)		12 (50.0%)*	1 (3.7%)*	<0.001
Ratio AST/ALT, median (range)		0.68 (0.35–1.03)	0.78 (0.47–1.67)	0.022
ALT quotient, median (range)		2.19 (1.03–4.15)	1.60 (0.15–4.90)	0.028
GGT quotient, median (range)		0.58 (0.22–1.80)	0.98 (0.18–2.50)	0.055
Forns index, mean ± SD		5.52±1.57	6.23±1.75	0.126
**Viral factors**				
nHap_E1E2, median (range)		17.00 (5–25)	21.00 (11–27)	<0.001
Absence of core 70R and 91L, N (%)		6 (24.0%)	16 (57.1%)	0.015
nHap_HVR1, median (range)		11.00 (2–17)	12.50 (1–20)	0.027
ISDR, N (%)				0.034
Wild-type		7 (28.0%)	7 (25.0%)	
Intermediate (1–3 mutations)		14 (56.0%)	21 (75.0%)	
Mutant (>4 mutations)		4 (16.0%)	0 (0%)	
Viral load (Log_10_IU/mL), mean ± SD		5.78±0.80	6.12±0.57	0.077
Ks_HVR1, mean ± SD		0.0744±0.0645	0.1077±0.0779	0.098

nHap_E1E2, number of haplotypes in the whole E1–E2 studied region; nHap_HVR1, number of haplotypes in the hypervariable region 1; ISDR, interferon-sensitivity determining region; SD, standard deviation; Ks, number of substitutions per synonymous site; ALT, alanine transaminase; AST, aspartate transaminase; GGT, gamma-glutamyl transferase; ×ULN, factor times upper limit of normal used in our center for males and females: 41 and 31 U/L for ALT, 37 and 31 for AST, and 85 and 50 for GGT, respectively.

* One missing value in each group.

### Baseline viral Variables Associated with Treatment Outcome

All patients were confirmed to be infected with HCV-1b by phylogenetic analysis of the core region (**Figure S2**). Amino acid composition analysis of this genetic region also showed that the absence of amino acid arginine (R) at position 70 and leucine (L) at position 91 was more frequent in non-responders (*p* = 0.015). Regarding the E1–E2 genetic variability estimates, while non-responders tended to have higher values than responders for most of the parameters, those most strongly related to treatment outcome were the nHap in the whole E1–E2 studied region (*p* = 4.23×10^−4^) and in the HVR-1 subregion (*p* = 0.027). The phylogenetic analysis of E1–E2 sequences confirmed the absence of contamination events (**Figure S3**). Regarding the ISDR region, all patients showing four or more mutations belonged to the responder group (*p* = 0.034). Finally, the viral load tended to be higher in non-responders (**Table 2**).

### Statistical Models for the Prediction of Treatment Outcome using Baseline Host and viral Variables

#### Discriminant analysis

The variables that persisted in the multivariate discriminant predictive model in decreasing order of significance were: nHap_E1–E2 (F ratio = 14.441), the core amino acid substitution pattern (F ratio = 12.219), the *IL28B* polymorphism (F ratio = 5.189), GGT ratio (F ratio = 4.623ALT ratio (F ratio = 1.696and viral load (F ratio = 0.774)This model was able to accurately predict the achievement of a sustained virological response in the training group (AUROC = 0.9444; 96.3% specificity, 94.7% PPV, 75% sensitivity and 81% NPV) when a 0.86 cut-off was used to maximize the PPV (**Table 3**). These values remained high when the model was applied to the validation group (AUROC = 0.8148, 88.9% specificity, 90.0% PPV, 75.0% sensitivity and 72.7% NPV). On the other hand, a 0.4 cut-off could be used to better predict non-response to treatment, maximizing the NPV (92% sensitivity and NPV, 85.2% specificity, and 84.6% PPV in the training group; 83.3% sensitivity, 80.0% NPV, 88.9% specificity, and 90.9% PPV in the validation group).

**Table 3:** Sensitivity, specificity, and predictive values for the predictive models obtained.

	AUROC (validated)	Sensitivity, % (validated)	Specificity, % (validated)	NPV, % (validated)	PPV, % (validated)
**Discriminant model**	0.9444 (0.8148)	75.0 (75.0)	96.3 (88.9)	81.0 (72.7)	94.7 (90.0)
**Decision tree**	0.9072 (0.7361)	95.2 (81.8)	84.4 (70.0)	96.4 (77.8)	80.0 (75.0)

AUROC, area under the receiver operating characteristic curve; PPV, positive predictive value; NPV, negative predictive value.

#### Decision tree analysis

**Figure 1:**
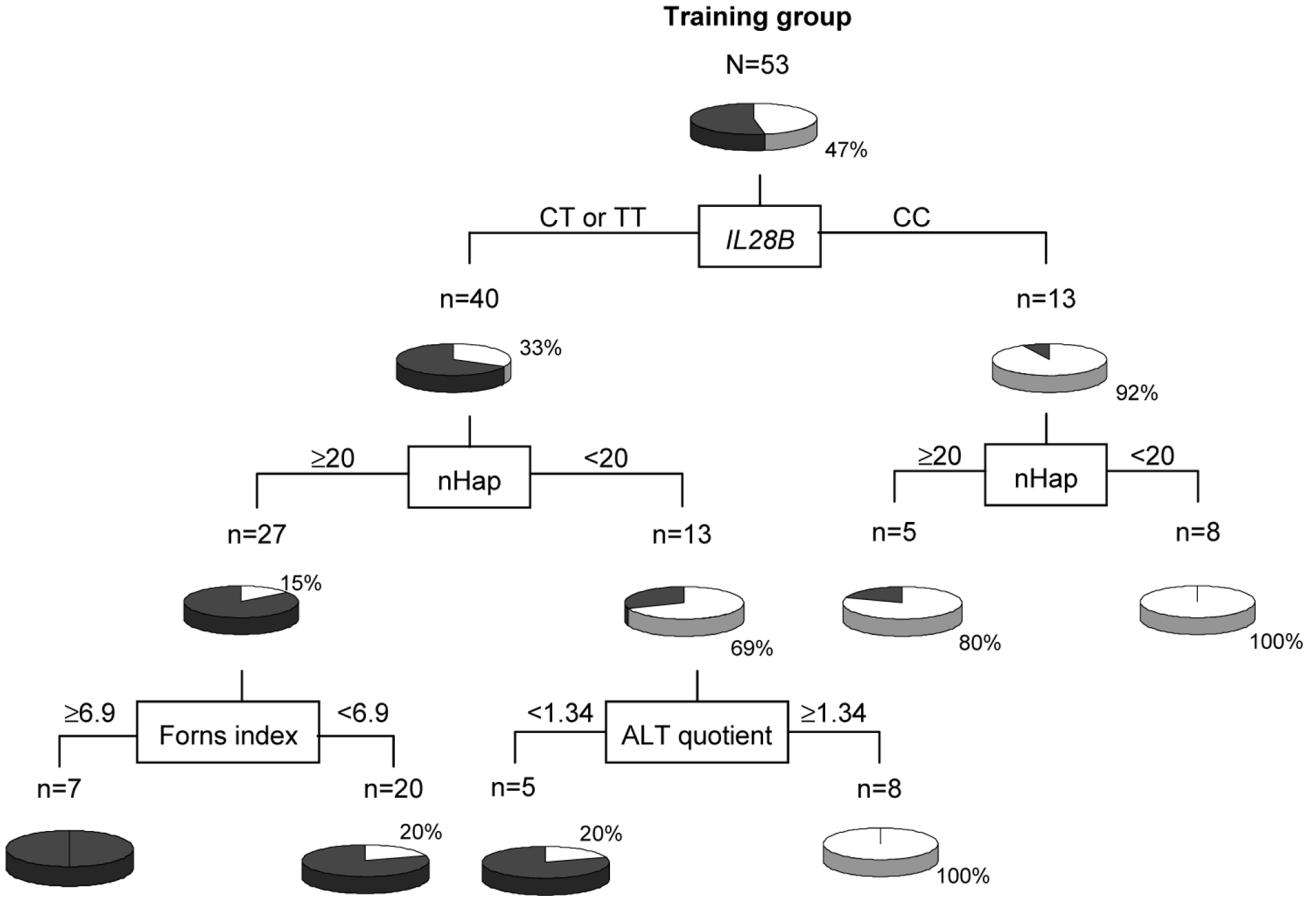
Decision tree model generated in the training group. The factors used for splitting and their cut-offs are indicated. Pie charts represent the rate of sustained virological response in white (the percentage is indicated) for each group of patients after each split. nHap_E1E2, number of haplotypes in the E1–E2 studied region; ALT quotient, square root of the alanine transaminase levels expressed as factor times upper limit of normal used in our center for males and females (41 and 31 U/L, respectively).

The generated decision tree is shown in **Figure 1**. The variables that persisted in this predictive model in decreasing order of significance were: the *IL28B* polymorphism (G^2^ = 14.1257), the ALT ratio (G^2^ = 12.8909), the nHap_E1–E2 (G^2^ = 12.1293), and the Forns index (G^2^ = 6.6038). This model was able to predict treatment outcome accurately in the training group (AUROC = 0.9072, 84.4% specificity, 80.0% PPV, 95.2% sensitivity and 96.4% NPV) (**Table 3**). In the validation group these values decreased to 70% specificity, 75.0% PPV, 81.8% sensitivity and 77.8% NPV (AUROC = 0.7361).

## Discussion

The new standard of care for chronic HCV-1 infection based on the administration of an HCV-specific PI, PegIFN-α and RBV has increased the treatment success rate [3]. However, this triple drug combination is associated with additional side effects and markedly higher health care costs than for PegIFN-α and RBV. It is important to consider that about 50% of HCV-1 patients successfully respond to the dual therapy [6], [7], which still is the current standard of care for HCV-1 chronic infection in many countries where PI are still not available or remain unaffordable. Moreover, in those countries where PI are already being administered, the triple therapy may not be appropriate for all patients; naïve patients with the *IL28B*-C/C genotype and F0–F2 fibrosis stage may still be treated with PegIFN-α plus RBV [26]. Therefore, a reliable prediction of response to dual therapy at baseline would be highly beneficial for the development of more effective and personalized treatment selection algorithms in order to optimize both patient wellbeing and health care expense.

### Predictive Models of Response to PegIFN-α and RBV Therapy

In this study, we developed two predictive models including host and viral variables that could help to improve treatment selection algorithms and assist clinicians in decision making.

The predictive model obtained by discriminant analysis generated an aggregate probability of response to treatment based on the *IL28B* polymorphism, and serum GGT and ALT levels as host variables, as well as the E1–E2 number of haplotypes, the core amino acid substitution pattern, and the viral load asviral variables. This model, which could be easily implemented in a computer-based application, showed an AUROC of 0.9444 and a high PPV both in the training and the validation groups (94.7 and 90.0%, respectively), thus offering a reliable prediction of SVR. As predictive models obtained by decision tree analysis might be easier to implement and interpret in the clinical setting, a second predictive model was generated. However, this model showed a lower PPV (80% and 75% in the training and validation groups, respectively) and a worse reproducibility than the discriminant one.

Other predictive models have been generated but only a few have been validated. To the best of our knowledge, those that have been developed for HCV-1b-infected patients showed a lower predictive accuracy than the ones described in this study. E. Martínez-Bauer *et al*. [27] developed a score based on multiple regression analysis including the AST/ALT ratio, cholesterol levels, the Forns index and the HCV viral load, and predicted SVR in a subgroup of patients with a high PPV (96% in the training group and 90% in the validation group); however, response could not be predicted in the group of patients with intermediate score values (50% of the total number of patients). M. Kurosaki *et al*. [28] developed a predictive model based on decision-tree analysis using the *IL28B* polymorphism, platelet levels, the viral load and the number of ISDR mutations, and predicted SVR with 78% sensitivity and 70% specificity. T. Takayama *et al*. [29] found that artificial neural networks analysis predicted SVR with more accuracy than regression analysis, and obtained a 59% sensitivity and 71% specificity based on a number of host variables and the HCV viral load. A. Tsubota *et al*. [30] developed a multiple regression model using the variables gender, age, platelet count, the *IL28B* and *SLC9A1* (a major ribavirin transporter gene) polymorphisms, and viral load, achieving a 73.3% PPV (71.4% in the confirmatory group). D. Miki *et al*. [31], using a prediction score based on multiple regression analysis including the variables BMI, *IL28B* polymorphism, and plasminogen activator inhibitor-1 (PAI-1) levels were able to predict SVR with 63% PPV (46% in the validation cohort).

### Relevance of Host Factors for Treatment Outcome Prediction

Among host-related factors associated with IFN-α-based treatment response, the polymorphisms upstream the *IL28B* gene constitute the strongest predictive factor of SVR identified so far [32]–[36]; however, European-American patients not having the most favourable *rs*12979860 genotype (C/C) still have approximately 40% chance of responding to therapy [34]. Similarly, this variable showed some limitations as a predictor in our study; among our population of Spanish patients only 33.3% were C/C, and while 87.5% of them responded to therapy, 31.3% of those who did not have this genotype also did. Consensus guidelines state that *IL28B* testing may be considered, but recommendations in favour of the use of this marker are not strong as its individual predictive value is low [3], [37].

While it is well established that patients with an advanced fibrosis stage respond worse than those with null or mild fibrosis, liver biopsy had only been performed for 35.1% of the patients and we had to rely on the non-invasive Forns index. Whereas this fibrosis indicator is able to reliably differentiate between patients with and without advanced fibrosis, intermediate stages are not classifiable, which may explain why this variable was not as strongly associated with treatment outcome as expected.

ALT levels were significantly higher in responder patients as previously reported [11], [38], while GGT levels were higher in non-responders. High GGT levels have been reported as an important independent predictor of treatment failure [39]–[41]. Higher GGT levels have been related to advanced fibrosis, steatosis and insulin resistance, which are more common among non-responders [42]. Furthermore, J. Everhart *et al.* suggested that GGT reflects a state of oxidative stress and that it should be regarded as a marker of disease activity, as GGT levels were found to predict both treatment response and liver disease outcomes [39].

We also took into consideration other variables that had been previously associated with SVR such as age, gender, BMI, AST/ALT ratio, and cholesterol, platelets and IP-10 levels. However, none of them persisted in the final predictive models. IP-10 seems to be associated with a stronger first-phase decline in the HCV viral load, and low levels of this chemokine have been associated with SVR [17], [43], [44]. However, other authors have found an association with rapid virological response but not SVR [45].

### Relevance of viral Factors for Treatment Outcome Prediction

The nHap_E1–E2 is an indicator of viral genetic heterogeneity, and a high value at baseline has been previously associated with dual therapy failure [14], [46], [47], either through the pre-existence or the generation of drug-resistant viral variants. This variable showed a greater significance in the discriminant predictive model than the rest of the variables. To the best of our knowledge, this is the first validated predictive model that includes a marker for baseline HCV quasispecies heterogeneity. While studying this variable by cloning and sequencing is labour intensive, alternative methodologies can be used; among them, next generation sequencing techniques have the capacity to simultaneously analyse several samples, which can decrease associated costs [48].

Baseline core amino acid substitutions at positions 70 (R by Q) and/or 91 (L by M) have been described as useful independent predictors of treatment failure in HCV-1b-infected patients [49]–[52]. However, this association has not been found in other studies [53] and it has been excluded from other predictive models [28], [54]. Several mechanisms have been proposed for the role of the Core protein in IFN resistance [55]–[57], and it has been suggested that this predictor could maintain its value in the era of triple therapy including Telaprevir [58].

A low HCV load has been suggested as a predictor of SVR [42], but the threshold to distinguish between low and high viral loads in not well established [37]. In our study, the viral load was treated as a continuous variable and, despite being marginally significant in the bivariate analysis, it was considered to be relevant in the discriminant model.

Finally, the association between the presence of ≥4 mutations in the ISDR and treatment response was initially described in Japanese patients [59] but it is less pronounced in European patients [60]. Only four patients in our study showed ≥4 mutations and all of them were responders, but this variable did not persist in any of the two generated models.

### Study Limitations

Our study has several limitations: (i) recent studies have suggested that other single nucleotide polymorphisms in several human genes are associated to treatment outcome in HCV-1-infected patients, including the human leukocyte antigen C (HLA-C) and the killer cell immunoglobulin-like receptor (KIR) genes [61], the programmed cell death-1 (PD-1) gene [62], and the inosine triphosphatase (ITPA) gene [63]. These polymorphisms were not considered in our study and could have increased the accuracy of the predictive models; and (ii) the sample size was limited to 74 patients given the laboriousness of the assessment of HCV genetic heterogeneity in the E1–E2 region. However, a similar number of patients were included in each group, accounting for the fact that about 50% of patients infected by HCV-1b achieve an SVR. In addition, we performed a validation of the obtained models in a comparable group of patients in terms of ethnicity, clinical background and HCV subtype.

### Conclusions

Achieving a rapid virological response at treatment week 4 has a high PPV (91%) for obtaining an SVR to PegIFN-α and RBV therapy, however, only 15–20% of persons with HCV-1 achieve this type of response [64], [65]. A sustained virological response to dual therapy could be predicted with a similarly high PPV (90.0% in the validation group) in our population of Spanish naïve HCV-1b-infected patients using the generated discriminant model, which was based on pretreatment host and viral variables. Those patients identified as responders could be treated with dual therapy with high chances of achieving an SVR; such a strategy could decrease the additional costs and side-effects associated with the triple therapy. Furthermore, most non-responders (88.9% specificity in the validation group) would also be identified as possible candidates for novel treatment regimens. Further studies should be performed to assess the applicability of the generated models to other populations.

## Supporting Information

Figure S1 **IL-10 levels in responder and non-responder patients in the training group.**
(PDF)

Figure S2 **Genotype 1 phylogenetic subtree of the core region.** Genotyped reference sequences available in the Los Alamos National Library HCV sequence database (http://hcv.lanl.gov/content/index) are shown in bold with the accession number and the HCV-1 subtype. The patients included in this study are identified with the patient identification number, accession number, and the treatment response group (R, responders; NR, non-responders). Substitution model: GTR+I+G (proportion of invariable sites: 0.369, gamma shape parameter: 0.449). Nodes supported with bootstrap values >70% (1000 replicates) are indicated. The scale bar represents substitutions per nucleotide position.
(PDF)

Figure S3 **Unrooted phylogenetic tree of the E1–E2 region.** All viral sequences obtained for each patient are identified with a vertical line, the patient identification number and the response group (R, responders; NR, non-responders). Substitution model: GTR+I+G (proportion of invariable sites: 0.311, gamma shape parameter: 1.094). All nodes corresponding to each individual patient were supported with bootstrap values >70%. The scale bar represents substitutions per nucleotide position. This tree shows the sequences derived from 31 patients; the phylogenetic tree for the rest of patients included in this study can be found at doi:10.1371/journal.pone.0014132.s001.
(PDF)
