# Supplementary figures and images for: Relevance of Baseline Viral Genetic Heterogeneity and Host Factors for Treatment Outcome Prediction in Hepatitis C Virus 1b-Infected Patients
Source: PLoS One. 2013 Aug 28;8(8):e72600. doi: 10.1371/journal.pone.0072600 (PMC3755994; doi:10.1371/journal.pone.0072600)

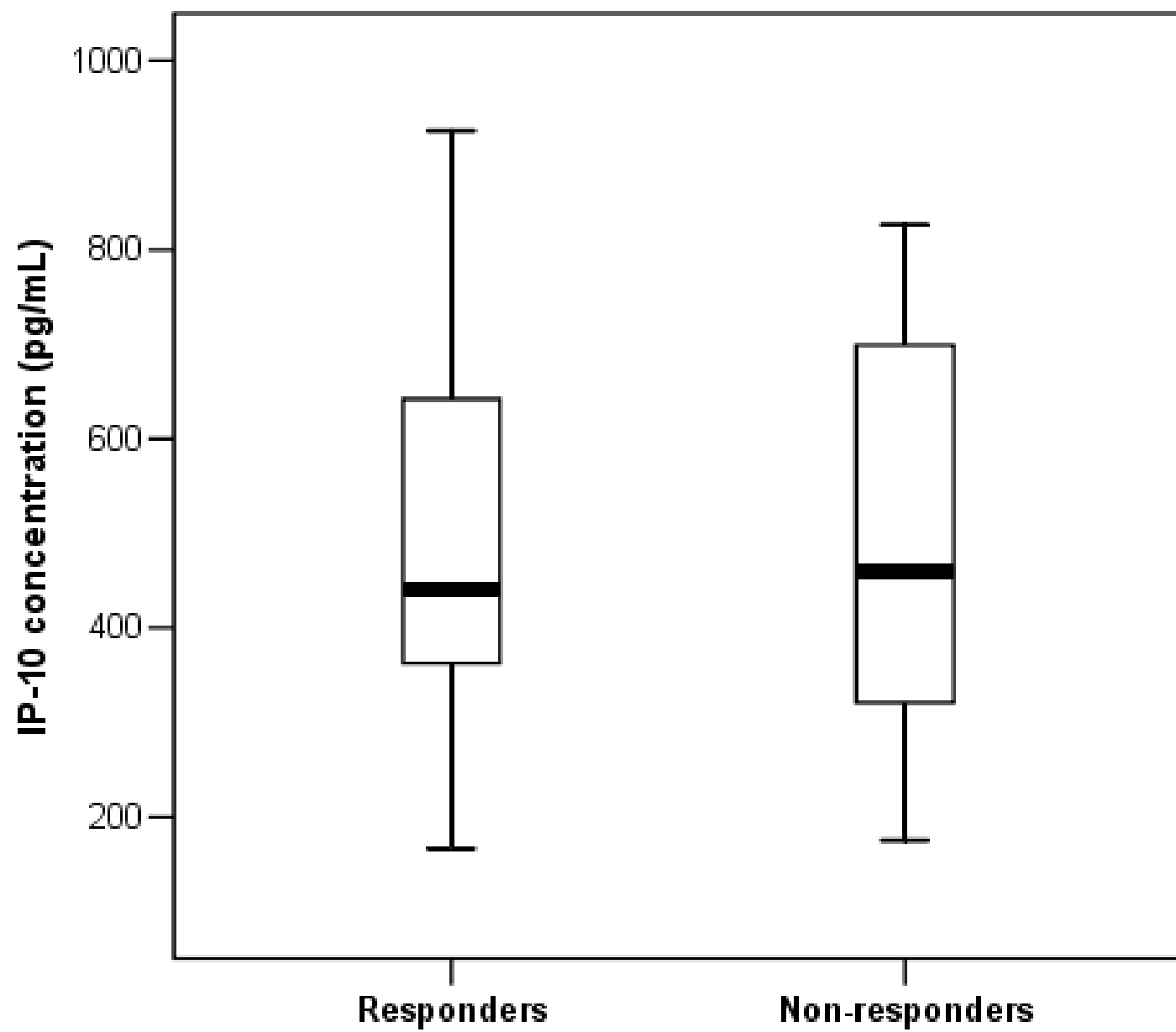

Supplement: Figure S1 — IL-10 levels in responder and non-responder patients in the training group. (PDF) [file pone.0072600.s001.pdf]

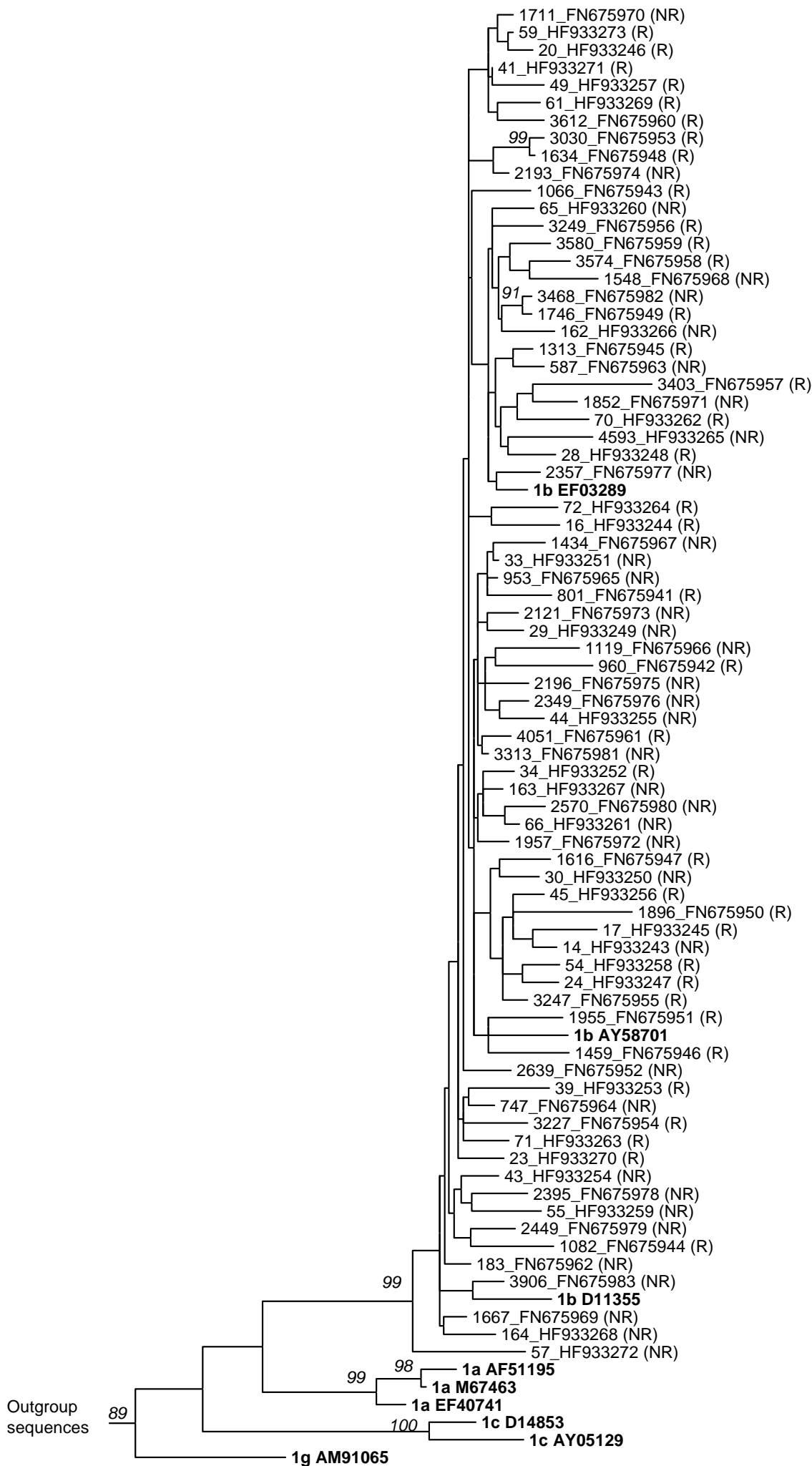

Supplement: Figure S2 — Genotype 1 phylogenetic subtree of the core region. Genotyped reference sequences available in the Los Alamos National Library HCV sequence database (http://hcv.lanl.gov/content/index) are shown in bold with the accession number and the HCV-1 subtype. The patients included in this study are identified with the patient identification number, accession number, and the treatment response group (R, responders; NR, non-responders). Substitution model: GTR+I+G (proportion of invariable sites: 0.369, gamma shape parameter: 0.449). Nodes supported with bootstrap values >70% (1000 replicates) are indicated. The scale bar represents substitutions per nucleotide position. (PDF) [file pone.0072600.s002.pdf]

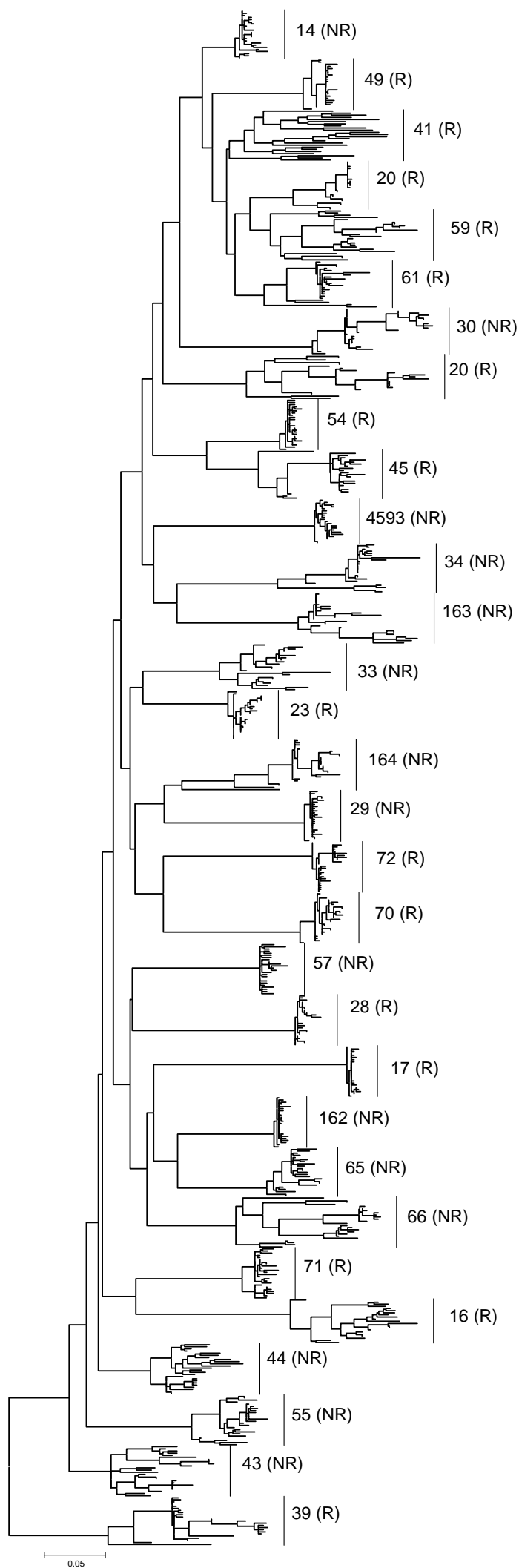

Supplement: Figure S3 — Unrooted phylogenetic tree of the E1–E2 region. All viral sequences obtained for each patient are identified with a vertical line, the patient identification number and the response group (R, responders; NR, non-responders). Substitution model: GTR+I+G (proportion of invariable sites: 0.311, gamma shape parameter: 1.094). All nodes corresponding to each individual patient were supported with bootstrap values >70%. The scale bar represents substitutions per nucleotide position. This tree shows the sequences derived from 31 patients; the phylogenetic tree for the rest of patients included in this study can be found at doi:10.1371/journal.pone.0014132.s001. (PDF) [file pone.0072600.s003.pdf]
